# Territory and population attributes affect Florida scrub‐jay fecundity in fire‐adapted ecosystems

**DOI:** 10.1002/ece3.9704

**Published:** 2023-01-15

**Authors:** David R. Breininger, Eric D. Stolen, Geoffrey M. Carter, Stephanie A. Legare, William V. Payne, Daniel J. Breininger, James E. Lyon, Chris D. Schumann, Danny K. Hunt

**Affiliations:** ^1^ Herndon Solutions Group, LLC, NASA Environmental and Medical Contract, NEM‐022 Kennedy Space Center Florida USA; ^2^ Department of Mathematics Florida Institute of Technology Melbourne Florida USA; ^3^ Merritt Island National Wildlife Refuge Titusville Florida USA

**Keywords:** conservation, hierarchical Bayesian model, long‐term studies, restoration, sociobiology, source‐sink, territory quality, zero‐inflated Poisson

## Abstract

Fecundity, the number of young produced by a breeding pair during a breeding season, is a primary component in evolutionary and ecological theory and applications. Fecundity can be influenced by many environmental factors and requires long‐term study due to the range of variation in ecosystem dynamics. Fecundity data often include a large proportion of zeros when many pairs fail to produce any young during a breeding season due to nest failure or when all young die independently after fledging. We conducted color banding and monthly censuses of Florida scrub‐jays (*Aphelocoma coerulescens*) across 31 years, 15 populations, and 761 territories along central Florida's Atlantic coast. We quantified how fecundity (juveniles/pair‐year) was influenced by habitat quality, presence/absence of nonbreeders, population density, breeder experience, and rainfall, with a zero‐inflated Bayesian hierarchical model including both a Bernoulli (e.g., brood success) and a Poisson (counts of young) submodel, and random effects for year, population, and territory. The results identified the importance of increasing “strong” quality habitat, which was a mid‐successional state related to fire frequency and extent, because strong territories, and the proportion of strong territories in the overall population, influenced fecundity of breeding pairs. Populations subject to supplementary feeding also had greater fecundity. Territory size, population density, breeder experience, and rainfall surprisingly had no or small effects. Different mechanisms appeared to cause annual variation in fecundity, as estimates of random effects were not correlated between the success and count submodels. The increased fecundity for pairs with nonbreeders, compared to pairs without, identified empirical research needed to understand how the proportion of low‐quality habitats influences population recovery and sustainability, because dispersal into low‐quality habitats can drain nonbreeders from strong territories and decrease overall fecundity. We also describe how long‐term study resulted in reversals in our understanding because of complications involving habitat quality, sociobiology, and population density.

## Graphical abstract

We conducted long‐term study to quantify how habitat, sociobiology, population factors, and random effects influenced Florida scrub‐jay fecundity to support adaptive fire management of an endangered species dependent on regular habitat disturbances. We used Bayesian hierarchical modeling of success (whether a family produced young) and counts (how many young) because many breeding pairs were unsuccessful in producing independent young during a breeding season.

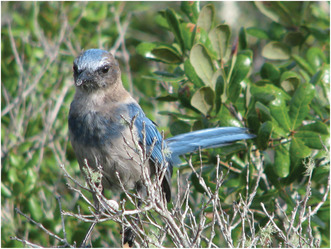

## 1 INTRODUCTION

Fecundity is fundamental for understanding fitness, population growth rates, conservation needs, and regulatory compliance, and for quantifying the effects of many ecological factors such as habitat, predation, food availability, weather, and population density (Martin, 1995, Ferrer & Donazar, 1996, Holmes, 2011). Fecundity is important to study the evolutionary advantages of group living in social animals (Koenig & Walters, 2015; Kokko & Ekman, 2002).

Many recent articles describe difficulties and misunderstanding when measuring fecundity accurately because fecundity results from more than one stochastic process (e.g., nest success, and fledgling survival) and because imperfect detection must be considered. Fecundity, sometimes described as productivity, refers to the number of young per breeding female at a period when young are approaching nutritional independence (Etterson et al., 2011; Schaub & Kery, 2021; Williams et al., 2002). Confusion and a misuse of terminology occur because other measures like the number of successful young per reproductive event (e.g., a successful nest attempt) and lifetime fitness are sometimes used to describe fecundity (Pincheira‐Donoso & Hunt, 2017).

The detection of all breeding females, nest attempts, and fledglings is difficult for many species and thus requires multiple models to properly account for imperfect detection (Etterson et al., 2011, Schaub & Kery, 2021). In some cases, a large proportion of breeders produce no young because of total nest failure or because no fledglings survive and analyses should consider zero‐inflated models recognizing that different ecological factors can influence each of the different processes (Brooks et al., 2017, 2019; Sofaer et al., 2014). Occasionally, a single, flexible model can deal with the overabundance of zeros (e.g., negative binomial or quasi‐Poisson), but such models sometimes lead to unstable and unreliable estimates (Ver Hoef & Boveng, 2007).

Surprisingly few studies of fecundity have been published using zero‐inflated models despite their widespread use in abundance and distribution models (Sofaer et al., 2014). Zero‐inflated modeling can use a Bernoulli submodel to study ecological factors (e.g., habitat, rainfall, population density) effecting nest failure and a Poisson submodel to the study factors effecting the counts of fledged young surviving to nutritional independence (Schaub & Kery, 2021; Sofaer et al., 2014; Zuur et al., 2009).

Here we study the fecundity of the Florida scrub‐jay, which is a habitat specialist endangered by deterministic and stochastic consequences of habitat loss, fragmentation, and the reduction in fire frequency relative to the natural fire regime (Chen et al., 2016; Coulon et al., 2010; Lacy & Breininger, 2021; Stith, 1999). Our objectives are to quantify how fecundity is influenced by habitat quality, presence/absence of nonbreeders, breeder experience, territory size, population density, and rainfall. Our objectives recognize that nest failure and fledgling mortality produce zeros and therefore we need to investigate the effects of environmental covariates separately on the Bernoulli process of nest success and the Poisson process where counts of young are influenced by survival after fledging (Kéry & Royle, 2015; Schaub & Kery, 2021; Sofaer et al., 2014).

### 1.1 Fires cause much spatial and temporal habitat heterogeneity

Our habitat quality objectives focus on distinguishing how fecundity differed among successional states, as such measures are needed in ecosystems maintained by periodic disturbances (Akcakaya et al., 2004; Akçakaya et al., 2005; Ellner & Fussmann, 2003; Hodgson et al., 2009; Meiman et al., 2020; Wilcox et al., 2006). Many ecosystems are subject to infrequent, large fires that result in territories with the same fire history within a landscape, but other ecosystems are subject to frequent, patchy fires that produce a fire mosaic among and even within territories (Bradstock et al., 2005; Driscoll, Lindenmayer, Bennett, Bode, Bradstock, Cary, Clarke, Dexter, Fensham, Friend, Gill, James, Kay, Keith, MacGregor, Russell‐Smith, et al., 2010). We have been refining how such habitat variation affects vital rates, at the scale of Florida scrub‐jay territories for decades to support annual state‐dependent habitat management decisions involving controlled fires (Breininger, Nichols, et al., 2010; Eaton et al., 2021; Johnson et al., 2011; Williams et al., 2011).

Fires affect the height of oak shrubs and abundance of open sandy areas, which are the key habitat determinants of Florida scrub‐jay vital rates in occupied territories. Here, we combine territories that are short or mostly tall into one territory quality state simply termed “sink” representing territories that burn too extensively or not enough. Our previous work showed that mortality exceeded recruitment in these habitats across a broad range of population densities (Breininger et al., 2006, 2009; Breininger & Carter, 2003; Breininger & Oddy, 2004).

Objectives regarding habitat quality here focus most on whether fecundity differs between two mid‐successional territory quality states made possible because of increasing samples following 30 years of habitat restoration. “Strong” territory quality is a combination of medium‐height oak scrub and open sandy areas and “weak” territory quality is dense medium‐height oak scrub. Distinguishing between strong and weak is important to identify the habitat conditions needed for long‐term population sustainability, as we appear to have incorrectly thought that weak could result in long‐term population sustainability (Breininger, Stolen, et al., 2014; Lacy & Breininger, 2021).

Strong is a management target important for many scrub plants and animals of conservation concern (Kent & Kindell, 2010; Woolfenden & Fitzpatrick, 1984). Strong is a difficult restoration target where openings persist for only 1–2 years after fire, especially because oaks take 3–8 years after fire to reach medium height (Duncan et al., 1995; Schmalzer, 2003; Schmalzer & Hinkle, 1992a, 1992b). Having both openings and medium‐height patches in close proximity occurs because Florida scrub‐jay territories are large (e.g., 10 ha at carrying capacity) and fires burn incompletely, producing mosaics at the territory scale with patches having different ages since the last fire (Breininger et al., 2018; Duncan et al., 2015). The great importance of such seemingly subtle fire effects often goes unrecognized in conclusions relevant to the sustainability of native fauna biodiversity (Clarke, 2008; Driscoll, Lindenmayer, Bennett, Bode, Bradstock, Cary, Clarke, Dexter, Fensham, Friend, Gill, James, Kay, Keith, MacGregor, Possingham, et al., 2010; Lindenmayer et al., 2008).

## 2 METHODS

### 2.1 Study species biology

Florida scrub‐jays are a medium‐sized passerine bird endemic to Florida scrub. For the first few years of life individuals typically remain in natal territories as nonbreeders and then disperse usually ≤2 territory widths away from their natal territories to breed (Breininger et al., 2006; Fitzpatrick et al., 1999). Florida scrub‐jays are cooperative breeders where nonbreeders assist parents in territorial defense, predator spotting and mobbing, and “helping” feed young in the nest and feeding newly fledged young (Fitzpatrick & Bowman, 2016; Mumme, 1992; Mumme et al., 2015; Woolfenden & Fitzpatrick, 1984). Florida scrub‐jay families maintain a sentinel system to spot predators and territorial intruders (Hailman et al., 1994; McGowan & Woolfenden, 1989). Clutch sizes are usually 3–5 eggs with 1–5 nest attempts per year because of great nest predation, and rare attempts to produce multiple broods except in areas with frequent supplementary feeding.

### 2.2 Study area

Our study areas consisted of 15 conservation areas along Florida's Atlantic coast (Breininger et al., 2006, 2009; Schmalzer et al., 1999) including Kennedy Space Center/Merritt Island National Wildlife Refuge, Sebastian River State Buffer Preserve, and numerous scrub reserves managed by the Brevard County Environmentally Endangered Lands Program. Habitat included oak scrub (e.g., *Quercus myrtifolia, Q. geminata*) on well or moderately drained sand ridges (old coastal dunes) in a matrix of lower elevation pine flatwoods (*Serenoa repens*, *Aristida stricta*, *Lyonia lucida*, *Pinus elliottii*, *P. palustris*) and marshes (e.g., *Spartina bakerii*). The ecosystem depends on fire as often as every 2–4 years with a mean return of 14 years (Duncan et al., 2009).

Habitat fragmentation reduced fire spread, and nearly all areas were subject to 20–30 years of active fire suppression followed by 10–40 years of controlled fires (Duncan et al., 1999; Duncan & Schmalzer, 2004). Controlled fires affect habitat differently than do natural fires partly because controlled fires occur under different seasons and meteorological conditions (Duncan et al., 2009). Once degraded by long periods without fire, scrub burns poorly under controlled conditions and requires mechanical cutting to prepare fuels; mechanical cutting is expensive and produces ecological effects different than fire by itself (Menges & Gordon, 2010; Schmalzer & Boyle, 1998). Restoration and maintenance of scrub produces heterogenous and uncertain responses because of annual rainfall, edge effects, fire history, and variability (Abrahamson et al., 2021; Breininger et al., 2018; Johnson et al., 2011). We use territory habitat quality state and not time‐since‐fire as a covariate because fires do not always kill all above‐ground oak stems and regrowth rates after fire are influenced by stored underground biomass and repeated fire frequency (Schmalzer & Foster, 2022).

### 2.3 Florida scrub‐jay field procedures

Our Florida scrub‐jay methods were similar to the 50‐year study at Archbold Biological Station (ABS) involving a comparably stable population in strong habitat (Chen et al., 2016; Fitzpatrick & Bowman, 2016; Woolfenden & Fitzpatrick, 1984). In contrast, nearly all our study populations declined by half due to degraded habitat quality from infrequent fire (Breininger et al., 1999, 2006).

We visited families at least once per month recording their bands and noting “hiccup” calls made only by females. Visits were weekly where we needed to band juveniles or unbanded immigrants. Adults and juveniles generally stayed together and flew to us to get peanut bits when we entered their territories, as described by Woolfenden and Fitzpatrick (1984). In April and May, we mapped territories by observing family disputes. Each bird was classified as a nonbreeding adult, breeder, or juvenile based on behaviors described by Woolfenden and Fitzpatrick (1984). Individual breeder and nonbreeder detection probabilities are typically above 0.9 and almost all members of the family remain close together (Breininger et al., 2009, 2022). We observed that nonbreeding adults often fed young (nestlings, fledglings, juveniles) and aided breeders in territorial defense, spotting and mobbing predators (Woolfenden & Fitzpatrick, 1984).

**FIGURE 1:**
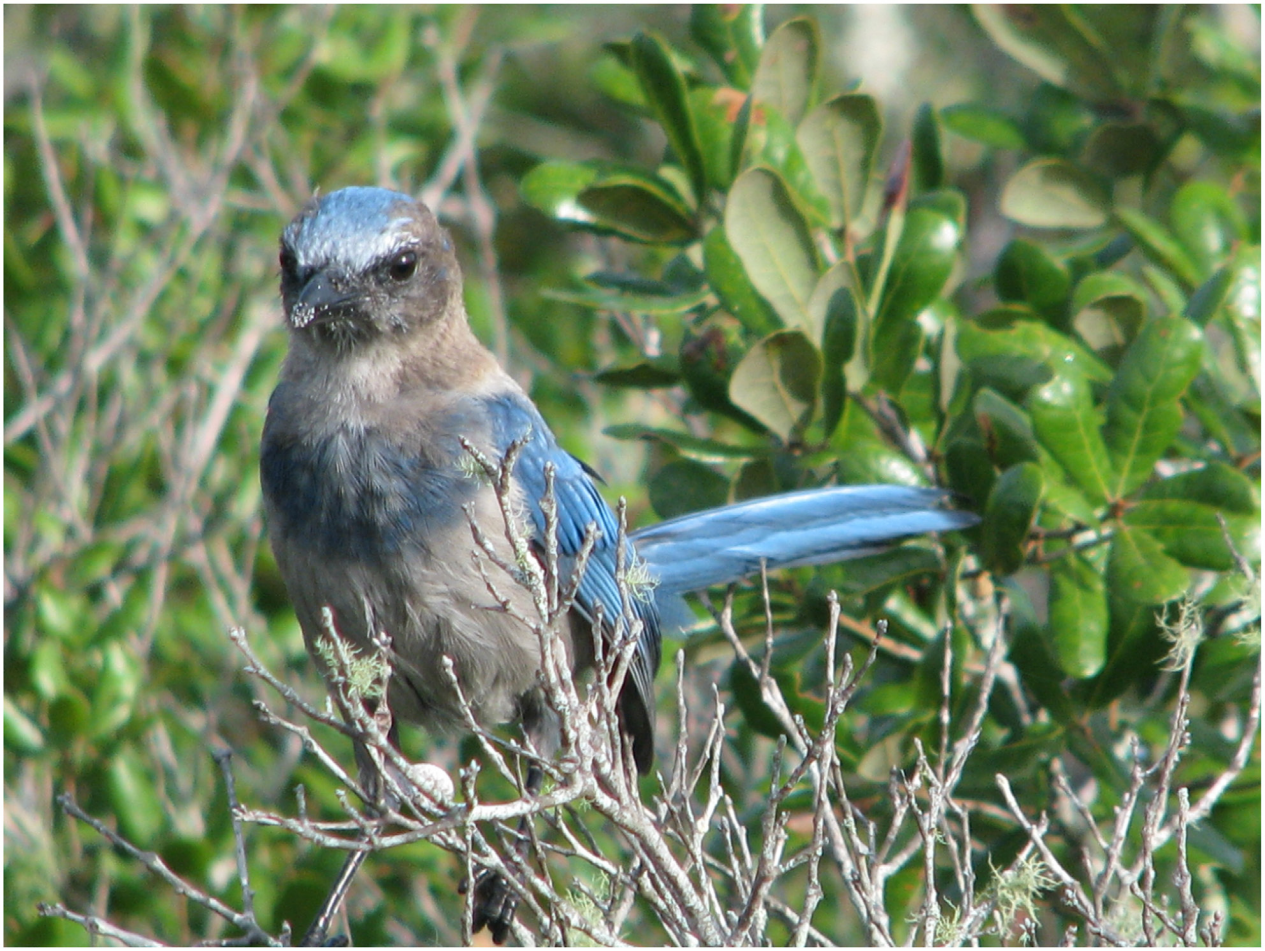
Juvenile Florida scrub‐jay with forehead starting to transition from a “brown head” into adult plumage being able to capture prey (e.g., insects) and fly with adults across the territory (e.g., ≥335 m width). The juvenile was within 15 m of all family members and had sand on the tip of the bill, while perching on 1.5 m tall scrub oak located along the edge of a sandy area that was within a one‐year‐old fire scar. The dense scrub oaks resprouted from underground biomass about 10 years before the 2nd last fire. Photo by David Breininger

Counts of juveniles in a territory each year were made in July as simple and efficient measurements of fecundity in contrast to the difficulty of finding and monitoring all nest attempts and fledglings in dense scrub (Carter et al., 2011). Juveniles in July are conspicuous and proficient fliers, following their families as they approach nutritional independence and have distinct brown heads compared to adults (Figure 1).

### 2.4 Dependent variable and individual data records

An individual data record referred to the annual number of juveniles counted in July in each territory (represented by one breeding pair); each record had associated covariates used to test hypotheses described below. Our predictions for β (regression) coefficients were summarized in Table 1. Years under investigation for individual study sites ranged from 3 to 31 years depending on funding, conservation acquisition, and permissions to access sites (Table 2).

**TABLE 1:** Comparing a priori predicted and observed regression slopes (β's) for Bayesian zero‐inflated Poisson model (having a success and count submodel) of Florida scrub‐jays juvenile production in east central Florida conservation areas, USA

*β* description	Predicted	Observed (mean)	SD	2.50% CI	97.50% CI	Rhat	n.eff
Success submodel							
Intercept		−1.11	0.19	−1.49	−0.74	1.00	2,477,204
Strong	Greatest +	1.28	0.15	0.98	1.59	1.00	198,151
Weak	+	0.34	0.14	0.07	0.61	1.00	2,390,511
Nonbreeders present	+	0.37	0.11	0.15	0.59	1.00	3.00 E+06
Population density	−	0.12	0.13	−0.14	0.38	1.00	1,180,318
Male experience	+	−0.07	0.11	−0.29	0.14	1.00	1,635,904
Female experience	+	0.07	0.10	−0.13	0.28	1.00	744,233
Territory size	+	0.20	0.15	−0.06	0.53	1.00	607,496
Mean rainfall	+	−0.22	0.33	−0.88	0.43	1.00	3.00 E+06
Count submodel							
Intercept		−0.41	0.24	−0.91	0.04	1.00	11,150
Strong	Greatest +	0.26	0.08	0.10	0.42	1.00	284,325
Weak	+	0.19	0.09	0.02	0.35	1.00	588,062
Nonbreeders present	+	0.10	0.06	−0.03	0.22	1.00	190,263
Territory size	+	0.08	0.09	−0.10	0.25	1.00	797,632
Mean rainfall	+	−0.01	0.12	−0.25	0.23	1.00	1,188,321
Proportion strong in population	+	0.18	0.09	0.01	0.36	1.00	287,400
Supplemental food in population	+	0.55	0.18	0.20	0.93	1.00	11,508
Population density	−	−0.14	0.09	−0.31	0.03	1.00	250,590
Random effects							
σ Year (success)		0.90	0.15	0.64	1.23	1.00	454,904
σ Year (count)		0.26	0.06	0.15	0.39	1.00	499,520
σ Territory (count)		0.06	0.05	0.00	0.17	1.00	447
σ Population (count)		0.18	0.08	0.05	0.37	1.00	36,698
Diagnostics							
Bayesian p‐value		0.41	0.49	0.00	1.00	1.00	868,297
Deviance		3626.93	75.05	3483.28	3777.58	1.00	788,355

*Note*: Rhat is the potential scale reduction factor (at convergence, Rhat = 1).

For each parameter. Effective sample size (n.eff) is a crude measure of effective sample size.

**TABLE 2:** The number of local Florida scrub‐jay populations and total territories studied varied by year in east central Florida conservation areas, USA

Year	Populations	Territories
1988	1	20
1989	2	37
1990	2	37
1991	2	36
1992	2	49
1993	2	45
1994	2	48
1995	2	58
1996	2	59
1997	5	94
1998	6	93
1999	8	99
2000	13	168
2001	14	218
2002	13	234
2003	7	190
2004	7	211
2005	7	182
2006	7	170
2007	7	185
2008	11	185
2009	3	91
2010	4	111
2011	4	104
2012	4	112
2013	4	121
2014	4	122
2015	4	116
2016	4	116
2017	4	104
2018	4	107

### 2.5 Covariate descriptions and hypotheses

We focused on comparing evidence for all covariates in one model and did not use model selection to eliminate covariates because we had information that all variables were important (Breininger et al., 2009; Breininger, Stolen, et al., 2014; Fitzpatrick & Bowman, 2016; Woolfenden & Fitzpatrick, 1984).

#### 2.5.1 Habitat quality at the territory and population scale

We characterized habitat quality states (strong, weak, sink) for each territory each year using field verification (Breininger et al., 2006, 2018; Carter et al., 2006; Eaton et al., 2021). Medium‐height was defined as having >0.4 ha oak scrub that was 1.2–1.7 m tall, but less than 0.4 ha tall scrub (>1.7 m). Here, we lumped all other habitat states as sinks because the scrub was too short or too tall or lacked enough scrub oak cover. As in the most recent studies, we classified the mid‐successional states in oak scrub as open‐medium (strong) or closed‐medium (weak) based on having ≥10 percent open sand within oak scrub patches (Breininger, Stolen, et al., 2014; Breininger et al., 2022; Eaton et al., 2021; Kent & Kindell, 2010). We hypothesized that strong had the greatest fecundity and that sink had the lowest when comparing territory quality states.

We hypothesized that the proportion of strong territories in each local population was an important population (group) covariate that increased fecundity. We hypothesized that a large proportion of strong territories made it easier for adults to spot, mob, and escape predators compared to sink areas having little escape cover or having many visual obstructions to conceal predators (Carter et al., 2007, 2011). We distinguished local populations based on whether there was >667 m (greater than two mean territory widths at carrying capacity) of unsuitable habitat between them given that 82%–92% of all dispersals remain inside such local population definitions (Breininger et al., 2006).

#### 2.5.2 Sociobiology at the territory scale

We hypothesized that the presence of at least one nonbreeding adult in a territory (family) increased fecundity by increasing both survival of nests and individual fledglings. The presence of nonbreeders (potential helpers) for a breeder pair was recorded as whether there was one or more nonbreeding adults present in the territory during April, which was the peak of the nesting season (Woolfenden & Fitzpatrick, 1984, Breininger, Carter, Legare, Payne, Stolen, et al., 2022). Fitzpatrick and Bowman (2016) found that the fecundity increases at a greater rate with low numbers of helpers and that fecundity decreases with high numbers. We fitted a post hoc model with a quadratic relationship of nonbreeders on juvenile counts (but not success) and found evidence for a nonlinear effect, but the parameters were poorly estimated (i.e., broad credible intervals of β's overlapping zero).

We hypothesized that experienced breeders increased fecundity in both submodels because experienced breeders were better at food provisioning, spotting predators, and competing for territories (Woolfenden & Fitzpatrick, 1984). Male or female breeding experience covariates were “1” if the male or female breeder was known to breed during an earlier nesting season; “0” for novice breeders; or “NA” if breeding experience was unknown.

Experienced breeders with nonbreeders are best able to compete for space and defend the largest territories (Fitzpatrick & Bowman, 2016). We also hypothesized that territory size increased fecundity in both submodels because larger territories were less likely to be burned completely (Fitzpatrick & Bowman, 2016; Woolfenden & Fitzpatrick, 1984). Large territories were likely to have more food, cover, and nest site opportunities that varied with time‐since‐fire. Territory size was recorded as the area in square meters calculated by digitizing territory boundaries into geographic information systems (e.g., Breininger et al., 1995, 2009).

#### 2.5.3 Local population covariates additional to habitat quality

We hypothesized that increased pair density of local populations decreased fecundity because of density‐dependent declines in demographic variables, previously observed in a population subject to a high immigration event due to nearby land clearing (Breininger & Oddy, 2004). Dense Florida scrub‐jay populations have strong competition for space and possibly food (Fitzpatrick & Bowman, 2016; Mumme et al., 2015). Density represented the number of breeding pairs divided by the number of potential territories in the local population (Breininger et al., 2006; Breininger, Carter, Legare, Payne, Stolen, et al., 2022). Potential territories were 10 ha grid cells that were of sufficient quality that they could be occupied despite whether recruitment exceeded mortality (Breininger et al., 1998; Burgman et al., 2001; Carter et al., 2006; Duncan et al., 1995). This approach was useful because populations declined greatly and territories often transitioned between sink and source states between years while remaining occupied (Breininger & Carter, 2003; Breininger, Nichols, et al., 2010).

Some Florida Scrub‐Jay populations experienced daily human feeding and we hypothesized such feeding increased fecundity (Schoech et al., 1997, 2004). We hypothesized an overall positive effect of supplemental feeding only where it occurred in conservation preserves. Supplementary food was assigned for populations in conservation areas bordered by bird feeders and where birds were regularly fed by human visitors, despite such feeding being unauthorized. We also hypothesized that local populations could have their own unknown random group effect not represented by the proportion strong, population density, and supplemental feeding because populations were bordered by different landcover types and were subject to different management histories in the past 60 years (Eaton et al., 2021).

### 2.6 Annual variation and random effects

Annual variation in the fecundity of Florida scrub‐jays in the study area has been large, and one explanation is that increased rainfall prior to nesting increases nest success because of greater food availability (Carter et al., 2011). Based on Carter et al. (2011) we included the cumulative rainfall 3 months (December, January, February) prior to the beginning of the nesting season as covariates on both the success and count submodels. The rainfall covariate varied at the year, but not the population or territory level. Rainfall data were obtained from the Global Historical Climatology Network for Titusville, Florida (Menne et al., 2012).

There is much unexplained annual variation in scrub‐jay recruitment and characterizing stochasticity is important in population analyses (Lacy & Breininger, 2021). We added a group random effect for year on both submodels, as is generally recommended in population analyses (Kéry & Schaub, 2011). We used random effects rather than fixed effects because we were not interested in a unique parameter to describe every specific year, population, or territory and the numbers of group sizes were large and would have a degrees of freedom cost. The choice of random effects can increase precision, allow broader interpretation of the relative importance of unobserved groups, and have many statistical benefits despite a slight (perceived) increase in model complexity (Gelman & Hill, 2006; Gomes, 2022; Kéry & Schaub, 2011; Schaub & Kery, 2021).

### 2.7 Territory random effects to address pseudoreplication and other concerns

We also included a random effect of territory on the count submodel to account for pseudoreplication concerns because long‐term data often include many of the same sites (unique patches of scrub), breeding pairs, individuals (e.g., dominant individuals), and genetic lineages across years and thus some group‐specific fecundity (Fitzpatrick & Bowman, 2016; Schaub & Kery, 2021; Woolfenden & Fitzpatrick, 1984). Territory identifiers were specific to a patch of scrub, typically having one or both breeders the same between years. We reasoned that a strong territory random effect would cause us to reconsider subdividing the territory random effect into additional group categories that might specifically include site, breeding pair, individual, or a measure of family lineage.

### 2.8 The success and count submodels combined to estimate fecundity

We developed a Bayesian zero‐inflated Poisson regression model of the number of juveniles produced in each territory. This model had a zero‐inflation process Bernoulli submodel (termed “success”) of whether a given territory and year produced ≥1 juvenile, and a juvenile count submodel (termed “count”) using a Poisson distribution. The success submodel was:

(1) $$S_{y,p,t}\sim \text{Bernoulli}\;\left(p_{y,p,t}\right)$$

where S_y,p,t_ was a binary variable equal to 1 if the group in year y, population p, and territory i successfully produced any young, and 0 otherwise. The effects of covariates on the success probability p_y,p,t_ were modeled on the logit scale as:

(2) $$\text{logit}\left(p_{y,p,t}\right)=\mathrm{\beta X}+{\text{Year}}_{p,t}$$

where β was a vector of betas for effects including an intercept term, X was the design matrix with the data including a leading column of all 1 s for the intercept. Year was a random effect for year modeled as:

(3) $${\text{Year}}_{p,t}\sim \text{Normal}\;\left(0,\sigma_{\text{year}}\right)$$

The success submodel was:

(4) $$C_{y,p,t}\sim \text{Poisson}\;\left(S_{y,p,t}\;\lambda_{y,p,t}\right)$$

where λ_y,p,t_ was the expected mean count for a group in year y, population p, and territory i. The effects of covariates on the expected mean count λ_y,p,t_ were modeled on the log scale as:

(5) $$\log\left(\lambda_{y,p,t}\right)=\mathrm{\beta X}+{\text{Year}}_{p,t}+{\text{Population}}_{y,t}+{\text{Territory}}_{p,t}$$

where β was a vector of betas for effects including an intercept term, X was the design matrix with the data including a leading column of all 1 s for the intercept, and Year, Population, and Territory were random effects modeled as:

(6) $${\text{Year}}_{p,t}\sim \text{Normal}\;\left(0,\sigma_{\text{year}}\right)$$

(7) $${\text{Population}}_{t}\sim \text{Normal}\;\left(0,\sigma_{\text{Population}}\right)$$

(8) $${\text{Territory}}_{t}\sim \text{Normal}\;\left(0,\sigma_{\text{Territory}}\right)$$

Bayesian models were fit using Markov Chain Monte Carlo (MCMC) methods using program JAGS 4.3.0 (Plummer, 2017) implemented in R (R Core Team, 2020) with the package jagsUI (Kellner, 2019) and visualized using R packages coda (Plummer et al., 2006), ggmcmc (Fernández‐i‐Marín, 2016), and Bayesplot (Gabry & Mahr, 2022).

### 2.9 Model implementation

We used uninformative normal priors with mean = 0 and SD = 10 (modeled on the logit scale for success and log scale for count submodel) for all model parameters, except for the priors representing the standard deviations for random effects. Weakly informative distributions were used for random effects by taking the right half of a normal distribution with mean 0 and SD = 2.24. This produced a distribution similar in shape to the Half‐Cauchy distribution recommended by Gelman (2006), but with a much shorter tail. The MCMC models with this distribution converged much faster because of the exponentiation of the random effect in the count submodel.

For each analysis, we ran 3 chains, initialized with different random starting values. We discarded the first 10,000 iterations as burn‐in, running additional samples until the Gelman‐Rubin convergence diagnostic (Rhat) was less than 1.01. The number of effective samples was estimated to be greater than 4000 for all parameters except for random effects which were much slower to converge. We did not thin the MCMC posteriors (Link & Eaton, 2012). All continuous and count covariates were scaled by subtracting the mean and dividing by 2 standard deviations to put the regression coefficients for categorical and numerical covariates on similar scales allowing comparisons of effect sizes (Gelman, 2008).

To evaluate evidence for model fit, we calculated a version of the Bayesian *p‐*value that compared the model fit of the actual data with the model fit of data simulated with estimated parameters (Kéry & Schaub, 2011). Within each MCMC iteration we calculated the Freeman‐Tukey statistic for the observed data and compared that with the statistic calculated for a new observation simulated using the estimated parameters. The Bayesian p‐value measured how extreme the data were compared with data simulated from the posterior distribution; values close to 0 or 1 indicated that predictions from the model rarely produced data like the actual observations.

### 2.10 Analyses of annual random effects (annual group‐level variables)

Because repeated good and bad years can decrease population viability (Burgman et al., 1993; Lacy & Breininger, 2021), we tested for patterns among years within the Bayesian paradigm. We calculated the number of times the sign changed in the sequence of estimated year random effect in each MCMC iteration, separately for success and count. Within each iteration, a random sequence of 0 or 1 was generated from a Bernoulli distribution of the same length. We then compared the posterior distribution of the number of runs for each random effect to that of the random Bernoulli sequence. The frequency that the number of runs in each year random effect was less than the number of runs (in the Bernoulli sequence) was tabulated as a Bayesian p‐value to measure evidence (probability) of a repeated pattern. This approach was similar to the runs test familiar in the frequentist paradigm (Wald & Wolfowitz, 1940).

We also calculated the correlation coefficient between the year random effects of success and count for each iteration and considered the correlation coefficient's posterior distribution as evidence for correlation between success and count values. We did this to describe whether the annual measures of uncertainty differed for both processes (e.g., nest success and survival of fledged young), hypothesizing that year‐level random effects were poorly correlated.

## 3 RESULTS

### 3.1 Model diagnostics

Seventy‐four percent of the pairs produced no juveniles during a breeding season confirm the importance of using a zero‐inflated modeling approach. Model convergence was achieved after running 3 chains for 1 × 10^6^ iterations, determined based on Rhat values <1.01. Effective sample sizes were >4000 and visual inspection of parameter histories and posterior distributions showed no problems with lack of convergence. The Bayesian p‐value was not near 0 or 1 and plots of residuals indicated no problems with the model fit.,

### 3.2 Effects of covariates in the success submodel

**FIGURE 2:**
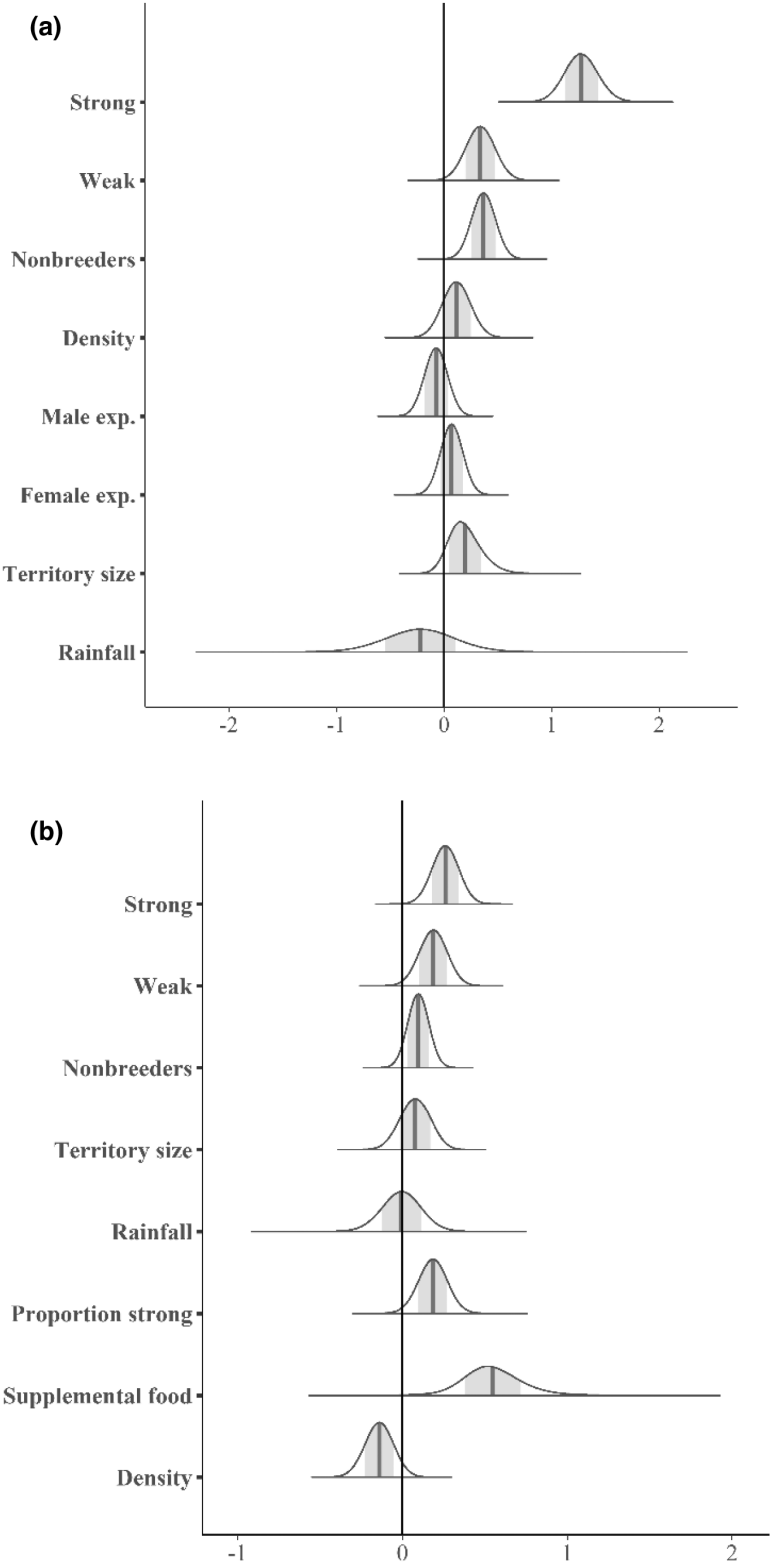
Posterior density (probability) plots for parameters in the Bayesian zero‐inflated Poisson model for Florida scrub‐jays in east central Florida conservation areas, USA, for the probability of success submodel (a) and the counts of juveniles produced per breeding pair submodel (b). In each plot the vertical bar shows the mean and the shaded area encompasses the 95% credible interval (CI).

**FIGURE 3:**
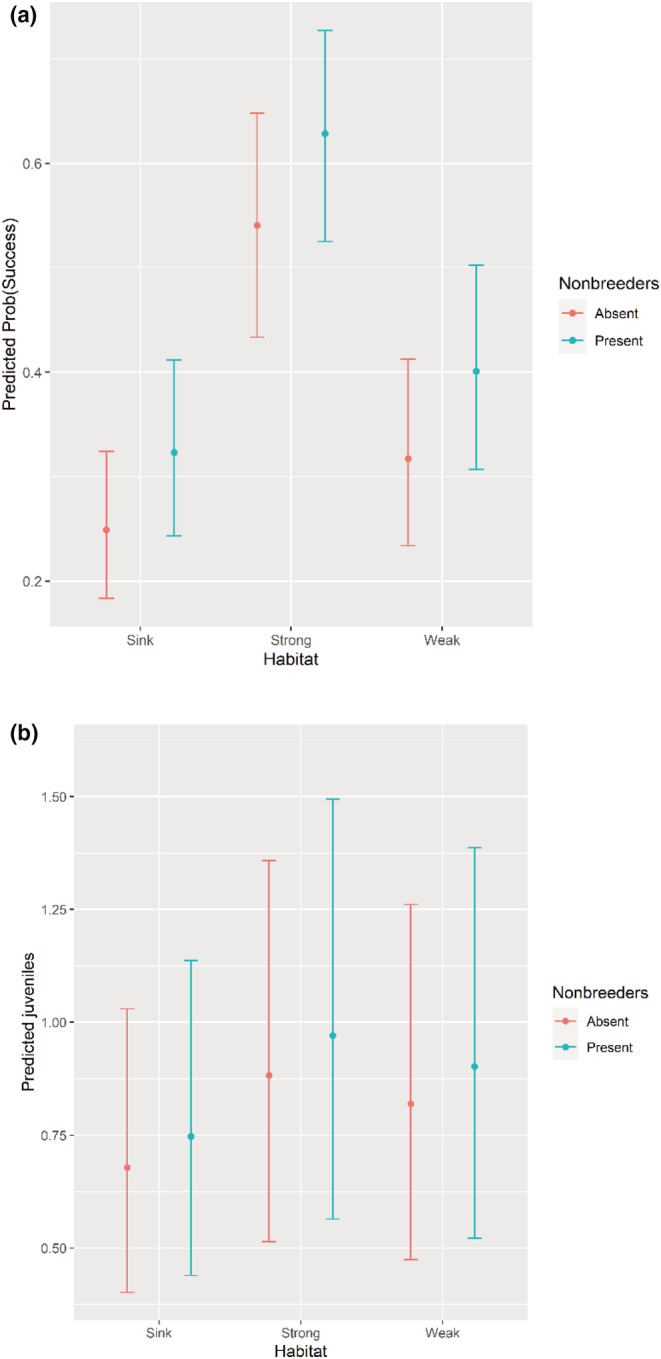
The predicted effects of the covariates on annual probability of success (a) and counts of juveniles produced per breeding pair (b) for the Bayesian zero‐inflated Poisson model for Florida scrub‐jays in east central Florida conservation areas, USA. The bars show the 95% credible interval (CI) for predicted means of all habitat state and nonbreeder combinations.

For the success submodel, territory habitat quality had the largest effect with strong having greater success than weak and weak having greater success than sink (Table 1, Figure 2). Pairs with nonbreeders (potential helpers) had a large positive effect on the probability of success (Figure 3). Territory size, population density, and female experience had effects in the predicted directions, but these parameter estimates had great uncertainty indicated by substantial portions of their posterior distributions overlapping zero. Although male experience and mean rainfall had estimated effects opposite of what was predicted, their posterior distributions (probabilities) broadly overlapped zero suggesting little to no support. Rainfall, a group (value) covariate for each year, explained little to no variation in success with the posterior distribution of the estimated effect broadly overlapping zero.

### 3.3 Effects of covariates in the count submodel

Supplemental feeding had the biggest effect on the counts of juveniles produced (Table 1, Figure 2). Territory habitat quality had the next largest effect with strong producing more juveniles than weak, but with a widely overlapping posterior distribution with weak (as a probability of uncertainty). The proportion of strong territories in populations also had a large effect on the counts of juveniles produced, and presence of nonbreeders (potential helpers) had a small positive effect. Territory size and population density both had effects in the directions predicted, but the posterior distributions broadly overlapped zero indicating poor precision in these estimates. Rainfall explained no variation in the count submodel.

### 3.4 Random and other unexplained effects on fecundity

**FIGURE 4:**
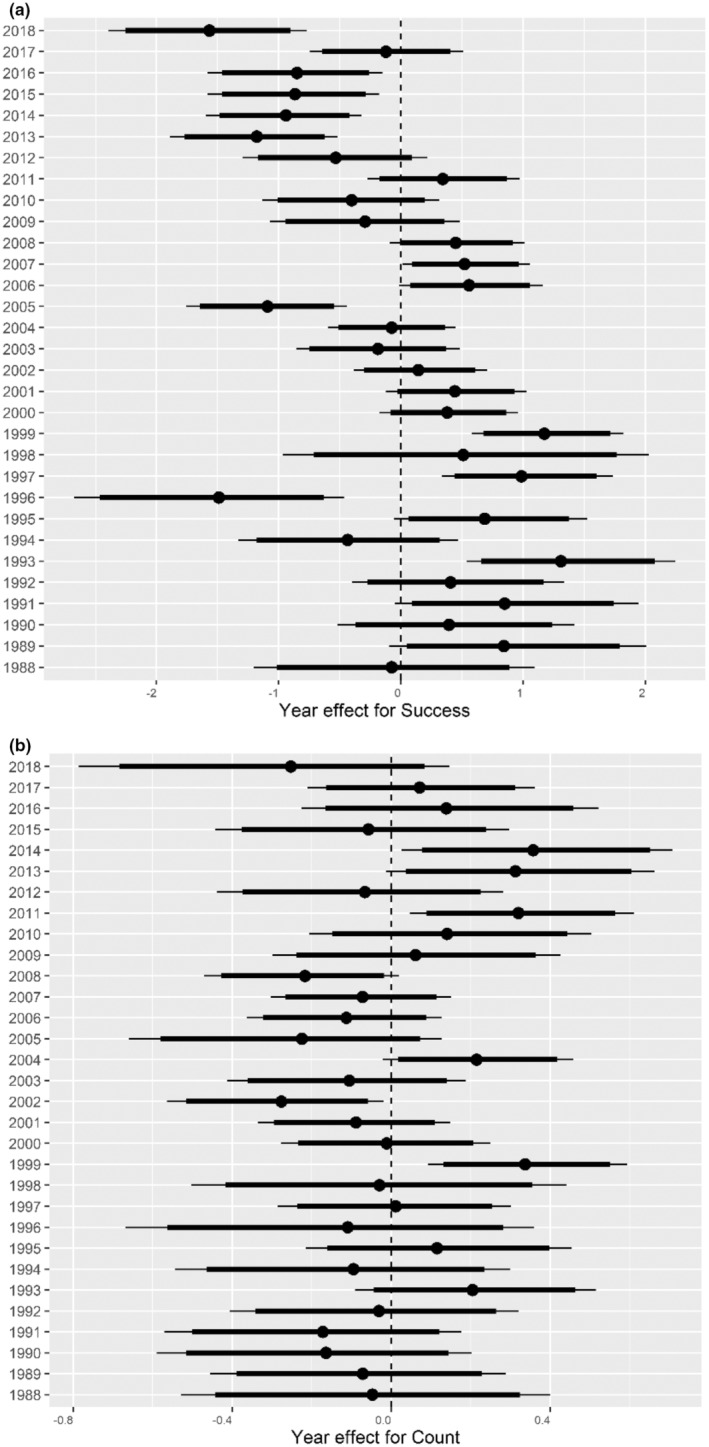
Annual highest posterior density intervals (HPD) of the random effect of year on success (a) and counts of juveniles produced per breeding pair (b) for the Bayesian zero‐inflated Poisson model for Florida scrub‐jays in east central Florida conservation areas, USA. The bars show mean, 90% and 95% credible intervals (CI).

The random effect of years (unknown effects of annual variation in fecundity) was important for both the success and count submodels (Figure 4). The predicted random effects of year for success and counts were not correlated (mean rho = −0.02, with a 95% CI (−0.30, 0.26)). We found slight evidence for sequences of good or bad years in nest success, but not counts of juveniles produced; as the Bayesian *p*‐value for the number of runs in success random effects was 0.14, and for counts was 0.36; values near 0 or 1 indicate evidence of patterns differing from random. The random effect for populations on count was important (local populations varied from each other for unknown reasons), although it explained less variation than the random effect of year. The random effect of territory on count was not important providing no evidence for pseudoreplication concerns (Table 1).

## 4 DISCUSSION

### 4.1 Habitat quality at the territory and local population scale

Habitat quality, represented by territory states and the proportion of strong territories in populations, was the most important deterministic factor influencing fecundity and matched a priori predictions. Understanding the effect of habitat quality on Florida scrub‐jay habitat preference, reproductive success, and survival is useful to inform adaptive management, especially to determine which successional state is needed for sustainable population growth (Breininger et al., 1995, 2009; Breininger, Duncan, et al., 2014; Eaton et al., 2021; Johnson et al., 2011; Williams et al., 2011). Understanding such habitat effects at multiple spatial scales is generally important in species management, as management actions at nest sites (e.g., protect nest trees) might differ from management actions of territory condition or actions across a larger unit of landscape (Purcell & McGregor, 2021; Reiley & Benson, 2019; Routhier et al., 2020). Our results support a management objective that aims for a large proportion of strong territories because only strong had fecundity rates able to sustain populations taking all habitat‐specific mortality rates and nonbreeder effects into account (Lacy & Breininger, 2021). Increased long‐term fecundity data combined with population models using all vital rates reversed our understanding because we incorrectly thought recruitment in weak was sufficient to sustain populations if nonbreeders were abundant (Breininger, Stolen, et al., 2014).

### 4.2 Importance of nonbreeders (helpers)

Pairs with nonbreeders had greater nest success than pairs without, and the effect in the nest success submodel was greater than in the count submodel. The number of nonbreeders increases fecundity in some cooperative breeders because of increased food provisioning to young and antipredator behavior (Griesser et al., 2017; Woxvold & Magrath, 2005). Fitzpatrick and Bowman (2016) found that increasing numbers of Florida scrub‐jay nonbreeders increased fecundity the most between 1–3 nonbreeders. A nonlinear effect might occur because nonbreeders enhance nest success, but become competitors for food as young get older (Mumme et al., 2015). We found evidence of a similar nonlinear effect, but with great uncertainty. Perhaps we did not find strong evidence that small, increasing numbers of nonbreeders had increased fecundity because sample sizes of more than one nonbreeder per family became low during long‐term population declines (Breininger et al., 2006).

### 4.3 Density dependence and population regulation

We found little to no evidence that pair density decreased fecundity suggesting other factors regulated the population. Pair density had small, opposite effects on success and count submodels and was poorly estimated, as posterior distributions of predictions (uncertainty) overlapped zero. We previously reported that many demographic variables (e.g., juvenile production, yearling recruitment, breeder survival) had weak negative correlations with pair density in a study site subject to an unusually large immigration event that produced unusually high pair densities for a few years (Breininger & Oddy, 2004). Perhaps social disruption of this large immigration event decreased demography, rather than increased pair density.

More recently, we found no evidence that breeder survival was correlated with pair density perhaps because of better predator detection associated with sentinel behaviors of many families. We also found evidence that nonbreeder survival actually increased with pair density, possibly because nonbreeders could monitor more territories for breeding vacancies across shorter distances putting themselves at lower predation risk. The benefits to group living provide many complications to density relationships among social animals (Griesser et al., 2017; Woodroffe et al., 2020).

Site‐dependent population regulation (Kluyver & Tinbergen, 1954; Rodenhouse et al., 1997) provided a more likely mechanism than density‐dependent decreases in vital rates for regulating Florida scrub‐jay population size. Such population regulation would occur because the proportion of sink territories would increase as pair density increased, leading to a lower average fecundity and declining population growth rate (Breininger & Oddy, 2004; Lacy & Breininger, 2021).

### 4.4 Other fecundity differences among populations

We found evidence that fecundity was greater in conservation areas with supplementary feeding. Food had once been thought to be sufficiently abundant for Florida scrub‐jays (Woolfenden & Fitzpatrick, 1984) and indiscriminate supplementary food was first thought to impact jays negatively by causing Florida scrub‐jays to nest before insects were seasonally abundant enough to support nestlings (Bowman & Woolfenden, 2001; Schoech et al., 2004). More recently, experimental and targeted supplementary feeding was reported to have management potential, especially during the early part of nesting when chicks need protein (Reynolds et al., 2003; Schoech et al., 2008). Supplementary feeding has been found to augment fecundity in other jay species (Derbyshire et al., 2015). Generally, further study of food quality and provisioning is needed to apply findings from food supplementation studies that have limited duration and scope (Benmazouz et al., 2021).

Here, we did not investigate food supplementation effects on small urban fragments because several studies found that mortality exceeded recruitment in urban areas due to a variety of other factors (Bowman & Woolfenden, 2001; Breininger, 1999; Mumme et al., 2000). We assume Florida scrub‐jay sustainability depends on the degree of habitat fragmentation; generally broad conclusions about edge effects and fragmentation levels need further replication and quantification across circumstances and species (de Satgé et al., 2019; Frantz et al., 2018; Stephens et al., 2004; White et al., 2020).

The proportion of strong territories, supplementary feeding, and pair density did not account for all group differences among populations, as we still observed random effects among local populations possibly because of factors not studied, such as predator–prey relationships and proximity to forests (Burgman et al., 2001; Carter et al., 2011). Forests have expanded with habitat fragmentation and associated reductions in the fire regime (Duncan et al., 1999, 2004; Duncan & Schmalzer, 2004). Increased forestation may have altered predator populations, especially yellow rat snakes (*Pantherophis alleghaniensis*) that prey on nests at night circumventing daytime predator vigilance and mobbing behaviors (Carter et al., 2007). The population random effect suggests limitations when using results from one population to predict ecological effects on other populations without replication.

### 4.5 Annual variation in fecundity

Our results suggested we still do not understand what drives much of the annual variation in fecundity in Florida scrub‐jays. An increasing number of studies show that animal populations are often limited by food abundance related to rainfall and other weather patterns (Boggs & Inouye, 2012; White, 2008). We found that rainfall had little effect and with a direction opposite to that predicted from an earlier study (Carter et al., 2011). The importance of rainfall and other weather factors often differs greatly among species and environments (Mangelinckx et al., 2020; Mattsson & Cooper, 2009; Saracco et al., 2016; Schöll & Hille, 2020).

We hypothesize that factors additional to total rainfall might explain annual variation, which can drive overall species population growth downwards (Burgman et al., 1993; Hilde et al., 2020); long‐term studies are generally needed to characterize the range of annual variation (Gaillard et al., 2000; LaHaye et al., 2004; Vincenzi et al., 2018). Population variation can be often largely driven by years with poor fecundity in small populations (Sæther et al., 2016).

Florida scrub‐jay populations are subject to severe, uncommon epizootic mortality events influenced by drought and rainfall cycling that also cause demographic variation. We predicted that success and count random effects would be lower in years after epizootic events in 1997 and 2002, but success was greater than average in 1998. Epizootic events also did not account for much of the annual survival variation (Breininger et al., 2009).

The annual fecundity patterns of a few successive good years or a few successive bad years can decrease population viability (Burgman et al., 1993) and might guide further research into the causes of annual variation. The slight support for sequences of good or bad years in random effects of the success model, suggested successive years are not entirely independent. We found that the annual random effects between success and count submodels were not correlated broadening support that these involved different processes. Evidence suggests that snakes are the primary nest predators and that mammals and hawks (e.g., *Accipiter cooperii*) become increasingly important as jays age (Breininger et al., 1996; Carter et al., 2007), but we have little information on how predator populations differ among years or populations.

### 4.6 Other covariates with no or little effect

We found that several covariates hypothesized to be important had little effect and were estimated poorly (e.g., broad credible intervals and coefficients overlapped zero). For example, female experience was mostly on the predicted side of zero but male breeder experience was on the opposite side, compared to ABS results (Woolfenden & Fitzpatrick, 1984). Territory size was an example where the posterior distribution was mostly on the predicted side of zero. Studies at ABS suggest territory size is positively correlated with fecundity (Grubb Jr et al., 1998; Mumme et al., 2015). Territory size might better explain fecundity variation within the long‐term ABS study area, which had mostly strong territories and a dense Florida scrub‐jay population in contrast to our study sites with varying territory habitat quality and lower population density. Perhaps the dynamic and spatial variation of territory habitat quality in our study sites had more influence than the effects of breeder experience and territory size.

Including territory random effects allowed us to account for repeated measures of breeding pairs and territories across years. We expected territory random effects given pairs or lineages had enhanced fitness at ABS (Suh et al., 2020; Woolfenden & Fitzpatrick, 1984). The strong effect of habitat early in our studies was often challenged as being possibly mistaken for other unmeasured effects, such as families with enhanced fitness. After decades of research across many sites, habitat quality states have remained influential; the low estimated variance for the territory random effect suggests that repeated measures concerns were comparably small. In our study sites, the spatial interspersion of strong, weak, and sink territories and annual transitions between states may have broken up the influence of repeated sampling of superior family groups making them less important than might be observed in a stable and less heterogeneous population such as ABS.

### 4.7 Importance of long‐term studies and many study sites

We observed fecundity rates in strong that were similar to those found for optimal (strong) habitat that dominated the long‐term study tract at ABS (Fitzpatrick & Bowman, 2016; Woolfenden & Fitzpatrick, 1984). Refining differences between strong and weak habitat was important because sample sizes for strong territories in our study sites had been increasing slowly because restoration can be expensive and is not always successful (Breininger et al., 2018; Breininger, Duncan, et al., 2014; Eaton et al., 2021; Johnson et al., 2011; Williams et al., 2011). Generally, repeated study refinement is necessary to characterize transient dynamics (Nichols et al., 2019) and long‐term data is often needed to address complications associated with habitat dynamics, sociobiology, and density dependence (Armstrong, 2005; Clutton‐Brock & Sheldon, 2010; Cosset et al., 2019; Hughes et al., 2017; Lindenmayer et al., 2012).

Most of our study sites remain dominated by sink habitat, which is common in other species occurring in dynamic landscapes because of anthropogenic changes (Aldridge & Boyce, 2007). The proportion of sink habitat quality is becoming of increased interest as the persistence of many globally imperiled species seems increasingly dependent on poor‐quality habitats (Kerley et al., 2020). The abundance of poor habitat quality (weak and sink) leads us to a new concern that many nonbreeders, which enhance fecundity, could be siphoned away to breeding opportunities in sink and weak territories reducing overall recruitment and population viability (Lacy & Breininger, 2021).

We earlier reasoned that weak and sink territories enhanced population persistence because these habitats can transition to strong, and birds preferentially move into strong habitat from sink after disturbances (extensive fires, epizootic events) keeping the strong territories filled (Breininger & Carter, 2003; Pulliam, 1988; Pulliam et al., 1992). However, habitat state transition rates vary greatly based on edge effects, fire history, and vegetation variables (Breininger et al., 2018) and having nonbreeders siphoned away by breeding opportunities in weak and sink could have an overall negative effect depending on the proportions of territory quality states available and breeding choices made by nonbreeders (Lacy & Breininger, 2021).

Further research is needed as nuances and even reversals in understanding occur as long‐term studies disproportionally improve the understanding of complex ecological processes (Clutton‐Brock & Sheldon, 2010; Hughes et al., 2017; Lindenmayer et al., 2012). Long‐duration studies and large sample sizes might be needed to target how the loss and formation of territories (thus population density of breeding pairs) is influenced by the complexities of habitat quality, breeder density, and sociobiology (e.g., mean family size, age, sex, relatedness among individuals). Nonbreeders generally fill annual vacancies associated with breeder deaths in occupied territories with little regard to territory quality and breeder population density (Breininger, Carter, Legare, Payne, Stolen, et al., 2022; Breininger, Stolen, et al., 2010), except in habitat unburned for many decades (Woolfenden & Fitzpatrick, 1984, 1991).

### 4.8 Conservation implications and summary

Long‐term data from many study areas demonstrated the need to increase the numbers and proportions of strong territories to improve fecundity, being of paramount importance for Florida scrub‐jay conservation regardless of complex ecological relationships. Managers often focus on habitat improvements in all areas; whereas we recommend managers prioritize limited resources on increasing the proportion of strong territories in areas where meeting recovery goals are most attainable and focus less on sink territories that are unlikely to transition towards strong. Long‐term restoration strategies lead to increasing proportions of many sink territories becoming strong, but we have observed few large increases in the proportion of strong territories using decades of data in most study sites and scrub‐jay conservation is approaching triage because many populations are becoming too small for long‐term sustainability (Breininger et al., 1999, 2006; Chen et al., 2016; Eaton et al., 2021; Johnson et al., 2011; Lacy & Breininger, 2021; Stith et al., 1996).

More generally, refining how fecundity varies among successional states is needed for sustaining the many declining grassland and shrubland organisms dependent on disturbance regimes modified by humans (Noss, 2013; Noss et al., 1995, 2015; Peterjohn & Sauer, 1999). The zero‐inflated Poisson modeling approach resulted in models that fit the data despite an overabundance of zeros resulting from more than one process, allowing investigation of the factors influencing the nest success and fledgling survival processes that together yield fecundity estimates (Schaub & Kery, 2021; Sofaer et al., 2014).

## AUTHOR CONTRIBUTIONS

**David R Breininger:** Conceptualization (lead); data curation (supporting); formal analysis (equal); investigation (equal); methodology (equal); software (supporting); writing – original draft (equal); writing – review and editing (equal). **Eric D Stolen:** Conceptualization (supporting); formal analysis (equal); methodology (equal); software (lead); validation (lead); writing – original draft (equal); writing – review and editing (equal). **Geoffrey Carter:** Conceptualization (equal); data curation (supporting); formal analysis (supporting); investigation (equal); methodology (equal); writing – original draft (supporting); writing – review and editing (supporting). **Stephanie A Legare:** Conceptualization (supporting); data curation (supporting); formal analysis (supporting); investigation (equal); methodology (supporting); writing – review and editing (supporting). **William V Payne:** Data curation (lead); software (supporting); validation (supporting); writing – review and editing (supporting). **Daniel J Breininger:** Data curation (supporting); investigation (supporting); methodology (supporting); writing – review and editing (supporting). **James Lyon:** Investigation (equal); methodology (equal); writing – review and editing (supporting). **Christopher D Schumann:** Data curation (supporting); formal analysis (supporting); software (supporting); writing – review and editing (supporting). **Danny K Hunt:** Data curation (supporting); formal analysis (supporting); software (equal); writing – review and editing (supporting).

## FUNDING INFORMATION

The study was funded by NASA Environmental and Medical Contract (NEMCON) Contract # 80KSC020D0023, US Fish and Wildlife Service, Florida Fish and Wildlife Conservation Commission, and Florida Department of Environmental Protection.

## CONFLICT OF INTEREST

Authors declare no conflict of interest.

## Data Availability

The data, R and Jags scripts are available at https://doi.org/10.6084/m9.figshare.21758609.
